# Optimizing passage, from primary care to hospital and back, for readmissions patients

**DOI:** 10.1186/1757-7241-20-S2-P36

**Published:** 2012-04-16

**Authors:** Poul Petersen, Jimmi S Olsen, Christian Skjærbæk, Thomas Nielsen

**Affiliations:** 1 Region Hospital Viborg, Medicinsk afd. Denmark; 2 Region Hospital Viborg, Karkirugisk afd. Denmark

## Background

Unplanned hospital readmissions are common, expensive and may be preventable. Strategies designed to reduce readmissions are necessary, especially in high risk patients. The purpose of this study was to conduct a follow up on medical and surgical patients, readmitted to A-24 Acute Ward (Regional Hospital Viborg, Denmark).

## Methods

A standardized questionnaire was used and the results were evaluated by an expert panel from the departments involved. To evaluate differences, we compared data from an audit held in 2010. The total number of patients was 933 in March 2010 and 937 in April 2011. The number of readmitted patients within 30 days was 14 in March 2010 and 37 medical and 24 surgical patients in April 2011. We evaluated all 14 patients from March 2010 and 20 random patients from April 2011. Medical records for these patients were retrospectively reviewed, and entered in an Audit questionnaire. In the audit team included the G.P. physician, home health care team, the patient and their relatives and the A-24 Acute Ward.

## Results

Within a month, the readmission rate was 1.5 % in 2010 and 6.5 % in 2011. The audit found three groups with special needs. Group 1: The psychiatric patients with addiction problems. Group 2: Cancer patients and Group 3: Aged multi morbid internal medical patients. Every group was represented equally.

## Conclusion

Several observations indicates that some readmissions can be avoided. These observations include significant variability in readmission rates in the different groups. General concerns were found in the inadequate follow-ups. One group of patients and relatives has complaints about inadequate preparation before discharge. Another concern was about poor doctor-to-doctor communication at the time of discharge. To reduce the hospital readmissions rate, we have implemented quality-of-care initiatives. Although the extent to which readmissions can be reduced is uncertain, reductions in the range of 20% to 30% are obtainable.

